# 

**Published:** 1892-01-02

**Authors:** 


No. 275, Vol. XI.—January 2nd, 1892.

[Registered at the General Post Office as a Newspaper for transmission in the United Kingdom and Abroad.]

WITH EXTRA NURSING SUPPLEMENT.
Per Annum, 6s. 6d., post free.
Monthly Parts, 6d.; post free, 8d.
Ditto per Annum, 8s., post free.

### THE HOSPITAL

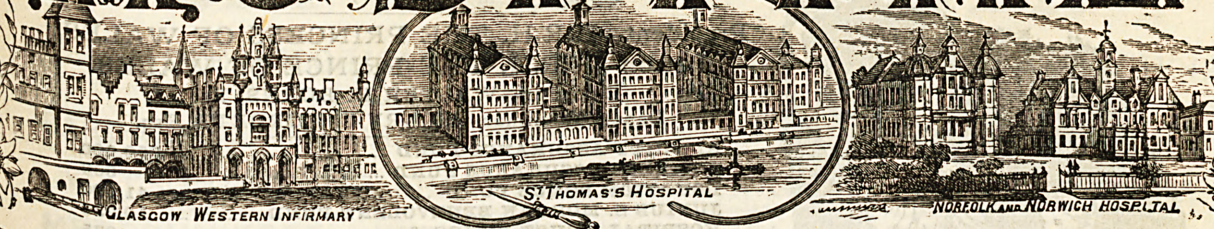

A WEEKLY JOURNAL OF

### Science, Medicine, Nursing, and Philanthropy.

SATURDAY, JANUARY 2nd, 1892.

London Voluntary Hospitals and their Critics - 161

Passing Topics.—The Jokes of Serious People - 162

1891 - 163

Everybody's Page - 165

Notes and News - 166

General Charities—Scraps and Gleanings - 167

Annotations.—London's Foggy Christmas—The Influenza—American Physicians on Virchow - 168

English Isolation Hospitals - 172

Funds and Fair-play for the Voluntary Hospitals of London - 173

The Practitioner's Mirror and Retrospect—

Medicine.—The Diagnosis and Surgical Treatment of Septic Thrombosis of the Lateral Sinus - 169

Surgery.—Multiple Cancellous Exostoses - 170

Practitioners' Bookshelf.—Photographic Group of the Bacteriological Section of the Congress of Hygiene - 171

New Drugs, Appliances, and Things Medical.—Condensed Milk - 171

The "Pall Mall Gazette" and the Hospitals - 176

The Student of Medicine.—Notes from the Schools—Students' Bookshelf—Appointments—Scholarships and Prizes.

EXTRA SUPPLEMENT.—THE NURSING MIRROR.

En Passant.—Nurses' Holidays—Keeping Christmas—Short Items—Nurses in Mashonaland—"The Year That's Awa'." - lxxix

A Nurse in Natal (Concluded) - lxxx

Leaves from a Nurse's Case-book - lxxxi

For Reading to the Sick—The New Year - lxxxii

Distributing Christmas Parcels - lxxxii

At Duty's Call - lxxxiii

Everybody's Opinion.—The Princess's Letter—Training for Nurses - lxxxiii

Appointments - lxxxiii

Off Duty.—Told on New Year's Eve - lxxxiv

Examination Questions - lxxxiv

Presentations—Amusements and Relaxation - lxxxiv

